# Timing of Nasogastric Tube Removal and Its Impact on Postoperative Bowel Function Recovery and Feeding Following Major Bowel Surgery in Children

**DOI:** 10.4314/ejhs.v36i1.8

**Published:** 2026-01

**Authors:** Ugochukwu Stanley Ezidiegwu, Agozie Nworah, AN Osuigwe, OH Ekwunife, JO Ugwu, Victor I Modekwe, CA Ugwunne, SO Obiechina

**Affiliations:** 1 Department of Surgery, Nnamdi Azikiwe University Teaching Hospital, Nnewi, Nigeria

**Keywords:** Abdominal surgery, children, early removal, nasogastric tube, postoperative care

## Abstract

**Background:**

Although studies in adults have shown that early removal of the nasogastric tube (NGT) after major bowel surgery is safe and more beneficial than delayed removal, adoption of this practice in children has been slow due to limited supporting evidence. This study aimed to compare postoperative outcomes between early and delayed nasogastric tube removal in children undergoing major bowel surgery.

**Methods:**

A total of 102 children who underwent major bowel surgery were randomized into two groups: Group I (early removal) and Group II (delayed removal), with 51 patients in each group. In Group I, the NGT was removed 24 hours postoperatively, whereas in Group II, it was removed after the passage of either faeces or flatus. The primary outcome was the prevalence of vomiting, while secondary outcomes included the timing of return of bowel sounds and the achievement of full-scale feeding. Data were analyzed using Statistical Product and Service Solutions (SPSS) version 20 (IBM, SPSS, Chicago, IL, USA). Qualitative and quantitative variables were analyzed using the Chi-square test and Student's t-test, respectively. A p-value < 0.05 was considered statistically significant.

**Results:**

Vomiting occurred in 6 (11.8%) patients in the early group and 5 (9.8%) patients in the delayed group (p = 0.750). Bowel sounds returned significantly earlier in the early group (83 ± 11.67 hours) compared with the delayed group (97 ± 17.71 hours; p = 0.029). Full-scale feeding was achieved at 121.84 ± 13.67 hours in the early group and 132.63 ± 12.79 hours in the delayed group (p = 0.031).

**Conclusion:**

This study reveals that early removal of the nasogastric tube might not significantly increase the rates of vomiting. However, it might lead to significantly reduced time to the return of bowel sounds and full scale feeding.

## Introduction

Following major abdominal surgery, there is a transient cessation of bowel motility known as postoperative ileus (POI), which typically lasts 3–5 days (1,2). After Levin introduced postoperative nasogastric tube (NGT) decompression in 1921, it became standard practice to decompress patients until the resolution of POI (3,4). Postoperative use of the NGT was also believed to facilitate earlier initiation of feeding and reduce hospital stay (4,5). This practice was widely accepted and passed down through generations of surgeons (4).

The precise mechanisms underlying POI are not fully understood. However, it is now evident that gastrointestinal motility returns earlier than previously believed—within 6–12 hours in the small intestine, 12–24 hours in the stomach, and 48–72 hours in the colon (2,6,7). Consequently, significant volumes of gastrointestinal contents may pass across anastomotic sites without disruption. These findings challenge the rationale for delayed postoperative nasogastric decompression based on the presumed prolonged duration of ileus.

One of the earliest studies supporting delayed NGT removal was conducted by Eade et al. in 1955 (8). Since then, numerous studies—primarily in adults—have evaluated this practice (4,9). Contrary to earlier assumptions, these studies demonstrated that early NGT removal shortens the duration of POI, allows earlier commencement of feeding, and reduces hospital stay (4,5,9). These findings have driven a shift toward earlier NGT removal following major abdominal surgery.

While adult studies have consistently demonstrated the benefits of early NGT removal, only a limited number of studies in children have reported similar outcomes (9). Given that children are not simply small adults, extrapolating adult findings to paediatric patients may be inappropriate or potentially harmful. Therefore, further paediatric-specific studies are required to validate the safety and benefits of early NGT removal in children.

Earlier feeding and reduced hospital stay may confer additional indirect benefits, particularly in low-resource settings. Malnutrition is endemic in Africa, and earlier initiation of feeding could mitigate its impact while improving wound healing and postoperative recovery (10). Furthermore, early NGT removal has been associated with shorter hospital stays, potentially reducing healthcare costs—an important consideration where approximately 85% of patients pay for care out of pocket (11). In developing countries such as Nigeria, limited paediatric surgical centres, personnel, and nursing resources further underscore the importance of practices that reduce workload and improve resource utilization (12,13).

This study compared the duration from the end of surgery to the return of bowel sounds and the time to achievement of full-scale feeding between children undergoing early versus delayed postoperative NGT removal.

## Methods

This prospective randomized study was conducted over a 24-month period from April 2020 to April 2022 at a tertiary health institution in Anambra State, Nigeria.

The study compared postoperative outcomes between early and delayed removal of NGT following major bowel surgery in children. Exclusion criteria included refusal of consent/assent, gastric or duodenal surgery, non-bowel surgery, unconscious patients, and those with deranged electrolyte values.

Ethical approval was obtained from the Research and Ethical Board of NAUTH. Written informed consent was obtained from caregivers prior to enrollment.

A total of 102 patients were recruited, with 51 patients allocated to each group. Participants were randomized preoperatively into Group 1 (early NGT removal) and Group 2 (delayed NGT removal) using simple random sampling with replacement via a ballot box.

Patients were recruited from the children's emergency unit, outpatient clinic, and paediatric surgical ward. Emergency cases were managed with nil per os, intravenous fluids, antibiotics, nasogastric decompression, urethral catheterization, and analgesics. Elective patients were intubated following induction of anaesthesia. All patients underwent routine investigations, including FBC and serum electrolytes, urea, and creatinine, and were optimized prior to surgery. The target preoperative haemoglobin level was ≥10 g/dL, and electrolyte abnormalities were corrected to at least the lower limit of normal.

Surgeries were performed by senior registrars or consultant paediatric surgeons. Anaesthesia was standardized, with induction using ketamine and midazolam, maintenance with isoflurane, and muscle relaxation achieved with atracurium. The end of surgery was defined as the time of placement of the final abdominal closure suture and served as the reference point for postoperative outcome measurements.

Postoperatively, patients received intravenous fluids, antibiotics, and analgesics, with close monitoring and correction of electrolyte abnormalities as needed. Caregivers were provided with data sheets to record vomiting episodes and passage of faeces or flatus.

Patients were reviewed twice daily. Data collected included vomiting frequency and characteristics, passage of faeces or flatus, and bowel sounds assessed by abdominal auscultation. The timing of these events was recorded and analyzed.

In Group 1, the NGT was removed 24 hours postoperatively. In Group 2, the NGT was removed after passage of faeces and/or flatus. Feeding was commenced after passage of faeces or flatus in both groups and advanced gradually until full-scale feeding was achieved.

Patients were discharged once discharge criteria were met, including stable vital signs, tolerance of feeding, satisfactory wound healing, absence of surgical site infection, and completion of intravenous medications.

Data were analyzed using SPSS version 20. Student's t-test was used to compare continuous variables, and p < 0.05 was considered statistically significant.

## Results

A total of 102 patients were enrolled and randomized equally between the two study groups. The study population comprised 50 females and 52 males. In the early group, there were 23 males and 28 females, while the delayed group included 27 males and 24 females.

The mean age of all patients was 27.31 ± 12.21 months (range: 1–62 months). There was no significant difference in mean age between the two groups (p = 0.082) (Table 1).

**Table 1:** Age distribution of the patients

Group	Mean Age (Months)	*p* Value
Early Group	30.82 ± 12.57	
Delayed Group	25.00 ± 11.23	0.082
Combined	27.31 ±9.12	

The mean duration of surgery was 104.46 ± 21.48 minutes, with no significant difference between groups (p = 0.317). Sex distribution was also comparable (p = 0.428).

Ileocolic intussusception was the most common diagnosis in both groups, accounting for 43.1% and 58.8% of cases in the early and delayed groups, respectively (Table 2). Right hemicolectomy with ileotransverse anastomosis was the most frequently performed procedure, with no significant intergroup differences.

**Table 2:** Indications for abdominal surgeries performed

Diagnosis	Early group	Delayed Group	*P* value
Colo-colic intussusception	3(5.9%)	2(3.9%)	
Ileo-colic intussusception	22(43.1%)	30(58.8%)	
Intestinal atresia	13(25.5%)	10(19.6%)	0.464
Malrotation Syndrome	13(25.5%)	9(17.6%)	

**Return of bowel sounds**: By postoperative day two, bowel sounds had returned in 13.73% of the early group and 3.92% of the delayed group. By day three, bowel sounds were present in 60.78% of the early group compared with 35.29% of the delayed group. The mean time to return of bowel sounds was significantly shorter in the early group (83 ± 11.67 hours) than in the delayed group (97 ± 17.71 hours; p = 0.029).

**Passage of faeces/flatus**: Passage of faeces or flatus occurred earlier in the early group, with a statistically significant difference between groups (p = 0.043). The mean time to passage was 96.79 ± 13.15 hours in the early group and 107.13 ± 15.38 hours in the delayed group (p = 0.041).

**Full-scale feeding**: Achievement of full-scale feeding occurred significantly earlier in the early group. The mean time to full-scale feeding was 121.84 ± 13.67 hours in the early group and 132.63 ± 12.79 hours in the delayed group (p = 0.013).

## Discussion

The patients in the early and delayed nasogastric tube (NGT) removal groups were comparable with respect to age, sex distribution, diagnosis, and surgical procedures performed. The mean age of patients in this study was 27.31 ± 9.12 months, which is younger than the mean ages reported in similar studies by Dinsmore et al. and Abantanga (14,15), where average ages were 7.2 and 6.33 years, respectively. This difference is attributable to variations in the predominant indications for surgery. In the present study, ileocolic intussusception constituted a large proportion of cases (55.9%), whereas ruptured appendicitis and gastrointestinal perforations were more common in the aforementioned studies. Intussusception remains a leading cause of emergency abdominal surgery in children, as reported in studies from Lagos and Enugu, Nigeria (16,17).

Return of bowel sounds is a recognized indicator of resolution of postoperative ileus. In this study, bowel sounds returned significantly earlier in the early NGT removal group compared with the delayed group (83 ± 11.67 hours versus 97 ± 17.71 hours, respectively; p = 0.029). Similarly, passage of flatus or faeces occurred earlier in the early group (96.79 ± 13.15 hours) than in the delayed group (107.13 ± 15.38 hours), with this difference also reaching statistical significance. These findings suggest that early removal of the NGT may facilitate faster recovery of bowel function following major bowel surgery in children.

Davila-Perez et al. (18) reported a shorter mean time to return of bowel sounds in both early and delayed groups compared with the present study, although the difference between their groups was not statistically significant. This discrepancy may be explained by the fact that all patients in their study underwent elective procedures, which are generally associated with less bowel handling and a shorter duration of postoperative ileus. Other paediatric studies did not include return of bowel sounds as a primary outcome, limiting direct comparison.

Achievement of full-scale feeding is a critical postoperative milestone. In the present study, full-scale feeding was achieved significantly earlier in the early NGT removal group (121.84 ± 13.67 hours) than in the delayed group (132.63 ± 12.79 hours). This finding is consistent with previous studies that demonstrated earlier tolerance of oral feeding following early NGT removal (19,20). The earlier attainment of full-scale feeding in the early group is likely related to the earlier resolution of ileus and earlier initiation of feeding following passage of flatus or faeces.

Earlier tolerance of full-scale feeding has important clinical and socioeconomic implications. It may contribute to improved nutritional status, enhanced wound healing, and faster postoperative recovery—particularly relevant in settings where malnutrition is prevalent (10). Additionally, earlier feeding and faster recovery may translate into shorter hospital stays, thereby reducing the overall cost of care. This is especially important in low-resource settings where a significant proportion of patients pay for healthcare services out of pocket (11). Furthermore, in environments with limited paediatric surgical centres, personnel, and nursing resources, early NGT removal could reduce caregiver burden, improve bed turnover, and optimize utilization of scarce healthcare resources (12,13).

Overall, the findings of this study indicate that early removal of the nasogastric tube following major bowel surgery in children is safe and associated with earlier resolution of postoperative ileus and earlier achievement of full-scale oral feeding, without an increase in postoperative vomiting.

This study had some limitations. Assessment of passage of flatus was challenging in younger children who could not reliably communicate this symptom. There was also potential inter-rater variability in the assessment of bowel sounds, as auscultation was performed by different research assistants. Minor variations in postoperative electrolyte levels, despite correction, may have also influenced bowel recovery. Larger multicentre randomized controlled trials are recommended to further corroborate these findings and strengthen the evidence base for routine adoption of this practice in paediatric surgical care.

## Figures and Tables

**Table 3:** Mean time to return of bowel sounds

Objective	Days postop	Early group	Delayed group	*P* value
	1	0	0	
	2	7(13.73%)	2(3.92%)	
Return of bowel sounds (Days)	3	24(47.06%)	16(8.10%)	
	4	12(23.53%)	17(33.3%)	0.042
	5	5(9.8%)	8(15.69%)	
	6	2(3.92%)	4(7.84%)	
	7	1(1.96%)	2(3.92%)	
	8	0	2(3.92%)	
Mean time to return of bowel sounds (hours)	---	83.7± 11.67	97.15 ± 17.71	0.029
